# Improving the contribution of mathematical modelling evidence to guidelines and policy: Experiences from tuberculosis

**DOI:** 10.1016/j.epidem.2024.100786

**Published:** 2024-09

**Authors:** C. Finn McQuaid, Nicolas A. Menzies, Rein M.G.J. Houben, Gabriella B. Gomez, Anna Vassall, Nimalan Arinaminpathy, Peter J. Dodd, Richard G. White

**Affiliations:** a TB Modelling Group, TB Centre and Centre for Mathematical Modelling of Infectious Diseases, Department of Infectious Disease Epidemiology, London School of Hygiene and Tropical Medicine, London, UK; b Department of Global Health and Population, Boston, MA, USA; c Center for Health Decision Science, Harvard T H Chan School of Public Health, Boston, MA, USA; d International AIDS Vaccine Initiative, Amsterdam, Netherlands; e Global Health Economics Centre, London School of Hygiene and Tropical Medicine, London, UK; f MRC Centre for Global Infectious Disease Analysis, Imperial College, London, UK; g School of Health and Related Research, University of Sheffield, Sheffield, UK

**Keywords:** Infectious disease modelling, Guidance, Quality and transparency, Tuberculosis

## Abstract

We read with great interest the recent paper by Lo et al., who argue that there is an urgent need to ensure the quality of modelling evidence used to support international and national guideline development. Here we outline efforts by the Tuberculosis Modelling and Analysis Consortium, together with the World Health Organization Global Task Force on Tuberculosis Impact Measurement, to develop material to improve the quality and transparency of country-level tuberculosis modelling to inform decision-making.

We read with great interest the recent paper by Lo et al. (2022), who argue that there is an urgent need to ensure the quality of modelling evidence used to support international and national guideline development. Good modelling evidence is difficult to generate, as disease and intervention mechanisms are frequently only partially understood; there may be a need to generalise evidence across settings and into the future, where trial evidence sometimes does not reveal implementation challenges, or only measures short or medium term outcomes. Indeed, the GRADE framework itself may not represent the optimal approach to including economic or modelling evidence in guideline development processes.

Ideally, ensuring good quality modelling evidence should take the form of an iterative process of continued engagement between modellers and guideline developers. Such an approach would allow for a continued improvement to modelling approaches, and subsequently strength of modelling evidence, rather than post-hoc rubber-stamping of evidence as either strong or weak (McQuaid et al., 2021). This need for improved modelling evidence extends beyond the development of international guidelines to the wider ecosystem of modelling to inform infectious disease policy. Much decision-making occurs after this step, for example resource allocation modelling to inform intervention optimization (TB Modelling and Analysis Consortium). Given similarities in the process of using modelling evidence to support decision-making, activities from the wider ecosystem could also be usefully applied to guideline development.

The Tuberculosis (TB) Modelling and Analysis Consortium, together with the World Health Organization (WHO) Global Task Force on TB Impact Measurement, have developed material to improve the quality and transparency in country-level TB modelling to inform decision-making. This material includes guidance for country-level TB modelling (World Health Organization and Modelling, 2018, Menzies et al., 2019) and benchmarking, reporting and review processes (McQuaid et al., 2021). These are focussed on the needs of countries making policy and funding decisions, however the optimal process for supporting guideline developers will likely be similar, as the majority of principles remain the same.

The guidance document describes 10 essential principles for country-level modelling, and associated good practices. These include principles of Relevance, Realism, Appropriateness of model structure, Consideration of all evidence, Validation, Informativeness, Transparency, Timeliness, Country ownership and Iteration. A flowchart, taken from the document, outlines the importance of these principles at each stage of a typical modelling project (see Fig. 1). Meanwhile, the benchmarking, reporting and review process operationalised this guidance through a set of quantitative benchmarks against which model assumptions and results could be compared, as well as a multi-stage review process with standardised reporting templates to provide feedback to modellers during the application and the users of modelling results after completion. This process was piloted in modelling applications in Kenya, Bhutan, Indonesia, Mongolia and Myanmar, where its use prompted important changes in the modelling applications as well as identifying wider issues affecting the production of modelling evidence, such as a lack of empirical evidence and capacity constraints. Elsewhere, the WHO Global Tuberculosis Report recently included the results of modelling estimates (World Health Organization, 2022), which similarly received external review to improve quality and transparency.

**Fig. 1:**
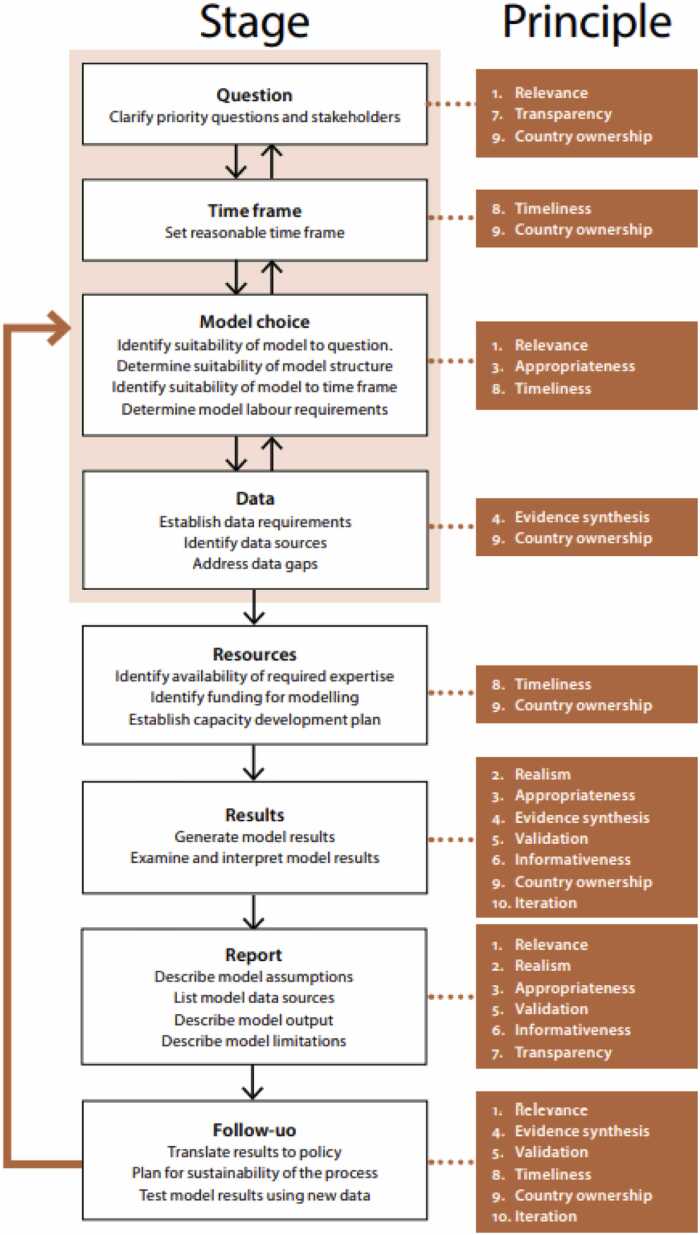
Flowchart of steps involved in a typical country-level modelling project.

A critical factor in driving the development of the above guidance and review process was a model comparison exercise for TB, held in 2015 (Houben et al., 2016, Menzies et al., 2016). Such exercises and ensemble modelling, with parallels in other disease areas such as HIV (Eaton et al., 2012), offer an alternative route to improving the robustness and contribution of modelling, highlighting uncertainty in model structure and parameters, and the consequences for the evidence produced. Similar exercises have compared the use of statistical models for subnational estimation of TB disease burden (Alba et al., 2022), or contrasted different approaches to modelling disease dynamics (Ragonnet et al., 2017, Menzies et al., 2018). While not necessarily explicitly aimed at guideline or policy development, these comparison exercises act to identify areas of concern and further strengthen good modelling practices, which should lead to improved modelling evidence and policy.

Ideally such reviews and comparison exercises would be routinely applied, to continuously improve the quality and transparency of modelling for decision-making. However, this requires both buy-in and incentives (in particular funding) for all of the key actors involved, including reviewers, evidence producers (modellers) and evidence consumers (such as the WHO, the Global Fund to Fight AIDS, Tuberculosis and Malaria, and country-level stakeholders). Despite significant advances in TB modelling to support decision making in recent years, a lack of funding to support continued implementation of these approaches remains a key risk to ensuring the quality of modelling evidence to inform guideline and policy development. Ideally this funding, which is comparatively cheap, should be included in modelling budgets for policy work routinely.

We wholeheartedly agree with the conclusions of Lo and colleagues (Lo et al., 2022) that further, sustained work is required to continuously improve and ensure the quality of modelling evidence, and commend their efforts to draw much-needed attention to this issue.

## Declaration of Competing Interest

The authors declare that they have no known competing financial interests or personal relationships that could have appeared to influence the work reported in this paper.

## References

[bib1] Alba S., Rood E., Mecatti F., Ross J.M., Dodd P.J., Chang S. (2022). TB hackathon: development and comparison of five models to predict subnational tuberculosis prevalence in Pakistan. Trop. Med. Infect. Dis..

[bib2] Eaton J.W., Johnson L.F., Salomon J.A., Bärnighausen T., Bendavid E., Bershteyn A. (2012). HIV treatment as prevention: systematic comparison of mathematical models of the potential impact of antiretroviral therapy on HIV incidence in South Africa. PLoS Med..

[bib3] Houben R.M.G.J., Menzies N.A., Sumner T., Huynh G.H., Arinaminpathy N., Goldhaber-Fiebert J.D. (2016). Feasibility of achieving the 2025 WHO global tuberculosis targets in South Africa, China, and India: a combined analysis of 11 mathematical models. Lancet Glob. Health.

[bib4] Lo N.C., Andrejko K., Shukla P., Baker T., Sawin V.I., Norris S.L. (2022). Contribution and quality of mathematical modeling evidence in World Health Organization guidelines: a systematic review. Epidemics.

[bib5] McQuaid C.F., Clarkson M.C., Bellerose M., Floyd K., White R.G., Menzies N.A. (2021). An approach for improving the quality of country-level TB modelling. Int. J. Tube Lung Dis..

[bib6] Menzies N.A., Gomez G.B., Bozzani F., Chatterjee S., Foster N., Baena I.G. (2016). Cost-effectiveness and resource implications of aggressive action on tuberculosis in China, India, and South Africa: a combined analysis of nine models. Lancet Glob. Health.

[bib7] Menzies N.A., McQuaid C.F., Gomez G.B., Siroka A., Glaziou P., Floyd K. (2019). Improving the quality of modelling evidence used for tuberculosis policy evaluation. Int. J. Tube Lung Dis..

[bib8] Menzies N.A., Wolf E., Connors D., Bellerose M., Sbarra A.N., Cohen T. (2018). Progression from latent infection to active disease in dynamic tuberculosis transmission models: a systematic review of the validity of modelling assumptions. Lancet Infect. Dis..

[bib9] Ragonnet R., Trauer J.M., Scott N., Meehan M.T., Denholm J.T., McBryde E.S. (2017). Optimally capturing latency dynamics in models of tuberculosis transmission. Epidemics.

[bib10] TB Modelling and Analysis Consortium. Country-level Modelling Applications [Available from: 〈https://tb-mac.org/tb-mac-resource/tb-modelling-roadmap-activities/〉. Accessed 19 January 2023].

[bib11] World Health Organization, 2022 Global Tuberculosis Report 2022. Geneva, Switzerland.

[bib12] World Health Organization, TB Modelling and Analysis Consortium, 2018. Guidance for Country-Level TB Modelling. World Health Organization, Geneva.

